# Impact of achondroplasia on Latin American patients: a systematic review and meta-analysis of observational studies

**DOI:** 10.1186/s13023-021-02142-3

**Published:** 2022-01-04

**Authors:** Virginia Fano, Chong A. Kim, Pablo Rosselli, Regina El Dib, Renée Shediac, Tatiana Magalhães, Debora Mesojedovas, Juan Llerena

**Affiliations:** 1 Hospital de Pediatría “Prof. Dr. Juan P. Garrahan”, Buenos Aires, Argentina; 2 Instituto da Criança HC – FMUSP, São Paulo, SP Brazil; 3 Fundacion Cardioinfantil-Instituto de Cardiologia, Bogotá, Colombia; 4 UNESP ‐ Univ Estadual Paulista, Department of Biosciences and Oral Diagnosis, Institute of Science and Technology, São José Dos Campos, SP Brazil; 5 BioMarin Pharmaceutical Inc, Novato, CA USA; 6 Medical Affairs Latin America, BioMarin Farmaceutica LTDA, São Paulo, SP Brazil; 7 Instituto Nacional Fernandes Figueira (IFF), Fundação Osvaldo Cruz, Av. Rui Barbosa 716, Rio de Janeiro, RJ 22250 020 Brazil

**Keywords:** Achondroplasia, Skeletal dysplasia, Latin America

## Abstract

**Background:**

Achondroplasia (ACH), the most common form of disproportionate short stature, is caused by a pathogenic variant in the fibroblast growth factor receptor 3 gene. Recent advances in drug therapy for ACH have highlighted the importance of elucidating the natural history and socioeconomic burden of this condition. Recognition that there are many potential issues for the patient with ACH is the first step in planning cost-effective interventions in Latin America (LATAM), a vast geographic territory comprising countries with multicultural characteristics and wide socioeconomic differences. We conducted a systematic literature review to characterize the impact of ACH on affected individuals and on healthcare resources in LATAM countries.

**Methods:**

Searches of the global medical literature as well as regional and local medical literature up to August 2020. Observational studies on patients with ACH from any LATAM country. Pairs of reviewers independently screened eligible articles, extracted data from included studies, and assessed their risk of bias.

**Results:**

Fifty-three unique studies (28 case series and cross-sectional studies and 25 case reports) including data on 1604 patients were eligible. Of these studies, 11 had data available for meta-analysis. Both premature mortality and all-cause mortality in the pooled studies was 15% [95% Confidence Interval (CI) 1.0E−3 to 0.47; I^2^ = 82.9%, *p* = 0.0029; three studies, n = 99 patients]. Frequency of cardio-respiratory-metabolic disorders was 17% [95% CI 0.04–0.37; I^2^ = 90.3%, *p* < 0.0001; four studies, n = 230 patients]; nervous system disorders was 18% [95% CI 0.07–0.33; I^2^ = 84.6%, *p* < 0.0001; six studies, n = 262 patients]; ear, nose, throat and speech disorders was 32% [95% CI 0.18–0.48; I^2^ = 73.4%, *p* = 0.0046; five studies, n = 183 patients]; and spinal issues including stenosis, compression and associated pain was 24% [95% CI 0.07–0.47; I^2^ = 91.3%, *p* < 0.0001; five studies, n = 235 patients].

**Conclusions:**

There is currently evidence of high clinical burden in ACH patients in LATAM countries. Establishing the impact of ACH provides the necessary foundation for planning tailored and effective public health interventions.

**Supplementary Information:**

The online version contains supplementary material available at 10.1186/s13023-021-02142-3.

## Background

Achondroplasia (ACH) is the most prevalent skeletal dysplasia, occuring with a frequency of 1 in 25,000 births [1–3], with a worldwide birth prevalence estimated to be 4.6 per 100,000 [4]. In Latin American (LATAM) countries, the birth prevalence ranges from 0.26 [4, 5] to approximately 0.45 [6, 7] in 10,000. However, data from the Latin American Collaborative Study of Congenital Malformations (ECLAMC) estimates a similar incidence in non-LATAM countries of 0.43 in 10,000 or 0.45 in 10,000 [8] 

In LATAM countries, ACH is often assumed to be compatible with a healthy and productive life. However, current evidence indicates that ACH is associated with a range of medical complications including obstructive sleep apnea, spinal stenosis, chronic pain, and cervicomedullary compression with subsequent risk of high surgical burden and death [9, 10]. In addition, ACH patients may experience a number of socioeconomic issues such as social isolation, lower self-esteem, less education, and lack of employment opportunities [11–15]. Management of all these complications can be challenging as it requires multidisplinary intervention.

Recognition that there are many potential issues for the patient with ACH is the first step in planning cost-effective interventions in LATAM, a vast geographic territory comprising countries with distinct cultures, socioeconomic structures, and public healthcare systems. However, due to the paucity of published studies on LATAM patients with ACH, particularly in the English-language medical literature, there is currently limited understanding of the impact of ACH on affected individuals and on healthcare systems in this region of the world. Moreover, of the published studies, the majority have stemmed from single centres with small patient cohorts. Furthermore, despite the growing evidence in the English-language medical literature of the clinical and psychosocial burden among patients with ACH, published studies are not exclusively based on LATAM populations and therefore the findings may not necessarily be generalizable to LATAM patients, and the experiences of living with ACH may not be the same across different regions of the world. We therefore conducted a systematic literature review and meta-analysis to better specifically characterize the impact of ACH in LATAM countries at the level of patient-important outcomes as well as at the economic (socioeconomic, healthcare utilization) level.

## Materials and methods

Our review followed recommendations for systematic reviews and meta-analyses (PRISMA) [16] of observational studies in epidemiology (MOOSE) [17] statements. This systematic review was registered in the PROSPERO (International Prospective Register of Systematic Reviews) database under the number CRD42020204963.

### Eligibility criteria

We included any epidemiological observational study (e.g., cohort, case–control, nested case–control, cross-sectional studies, prospective case series, case report) on patients with ACH from any LATAM country (e.g., Brazil, Argentina, Colombia, Mexico, Costa Rica, Peru), regardless of whether they reported our pre-defined patient-important outcomes and/or economic burden outcomes defined below. A diagnosis of ACH in patients in the included studies was based on genetic confirmation and/or clinical diagnosis of ACH (clinical examination and/or radiological assessment).

We excluded studies that evaluated patients with only hypochondroplasia as well as commentaries, reviews, off-topic studies, and those with co-occurrence of ACH and another syndrome.

For patient-important outcomes, we were interested in investigating the following:

- Mortality:
  - Premature mortality defined as sudden death within 1 year of age;
  - All-cause mortality; and
  - Cardiovascular mortality.
- Physical comorbidities:
  - Cardio-respiratory-metabolic disorders (e.g., cardiovascular diseases, obstructive sleep apnea, obesity).
  - Nervous system disorders (e.g., cervicomedullary compression, gross motor delay);
  - Ear, nose, throat and speech disorders (e.g., otitis media, hearing loss, upper airway obstruction, speech delay);
  - Spinal issues including stenosis, compression and associated pain (e.g., chronic back pain, symptomatic spinal stenosis, thoracolumbar kyphosis, lumbar hyperlordosis);
  - Orthopedic complications (e.g., chronic leg pain, wheelchair bound, limited elbow extension);
  - Pain;
  - Perinatal complications (e.g., premature birth, hospitalization); and
  - Others (e.g., hypothyroidism, difficulty in performing epidural anesthesia for cesarean delivery, tumours, blood transfusion, length of hospitalization).
- Humanistic:
  - Psychosocial disorders: depression, anxiety, bullying, isolation, hopelessness, somatization, humiliation, stigma, perception about their psychosocial life, etc., measured by non-validated and validated questionnaires as defined by the included studies;
  - Delayed self-care skills (e.g., toileting, cup-drinking);
  - Suicide attempts, and/or suicide rates; and
  - Social adaptation challenges;
  - Impact of the disease on patient and/or caregiver health-related quality of life, activities of daily living, work productivity, education, employment, social, and so forth; and
  - Quality of life measured by non-validated and validated questionnaires, as defined by the included studies, such as the Brief Pain Inventory-Short Form (BPI-SF) Questionnaire, the Quality of Life Short Stature Youth (QoLiSSY) Questionnaire, and the Pediatric Quality of Life Inventory (PedsQL).

At the economic level, we were interested in investigating the following outcomes:

- Socioeconomic burden (e.g., securing employment) measured by non-validated and validated questionnaires, as defined by the included studies such as the Work and Productivity and Activity Impairment (WPAI-SHP).
- Environmental burden:
  - Lack of equipment, furniture, toys, shoes, etc., matching anthropometric limitations;
  - Limitation of physical access to transportation modalities;
  - Adaptation to standard transport equipment and;
  - Challenges in physical activity.
- Health economic impact:
  - Direct and/or indirect costs, treatment costs, health care resource use, cost of comedications, hospitalizations.

### Data source and searches

Using Medical Subject Headings (MeSH) based on the terms “achondroplasia” and “skeletal dysplasia” (Additional file 1: Table S1) we performed the search in the global medical literature using the Medical Literature Analysis and Retrieval System Online (MEDLINE, via PubMed, from 1946 to August 2020), Excerpta Medica Database (EMBASE, via Elsevier, from 1974 to August 2020), Cochrane Central Register of Controlled Trials (CENTRAL, via Wiley, issue 8, 2020), and Web of Science (to August 2020).

We also conducted the search using both Spanish and English terms in the regional and local medical literature using Latin American and Caribbean Health Sciences Literature (LILACS, 1982 to August 2020), Scientific Electronic Library Online (SciELO, 1997 to August 2020), SciVerse Scopus via Elsevier (to August 2020), the Spanish Bibliographic Index of the Health Sciences (IBECS, 1983 to August 2020), National Bibliography in Health Sciences Argentina (BINACIS, to August 2020), Caribbean Health Sciences Literature (MedCarib, to August 2020), National Medical Sciences Information Center of Cuba (CUMED, to August 2020), and the Brazilian Bibliography of Dentistry (BBO to August 2020). The date of the last search was August 18, 2020.

We also searched the gray literature including ProQuest Dissertations & Theses Global (1989 to 2020), the National Health Surveillance Agency (ANVISA), Brazilian Digital Library of Theses and Dissertations (BDTD), Latindex Redalyc Latam, Mexico National Institute of Pediatrics website, and conference proceedings. In addition, reference lists of relevant primary studies were hand searched and experts in the field were contacted to obtain additional unpublished data.

We did not impose any language or year restrictions. The search strategy was adapted for each database to achieve more sensitivity. Duplicate records across databases were removed.

### Selection of studies

**Fig. 1:**
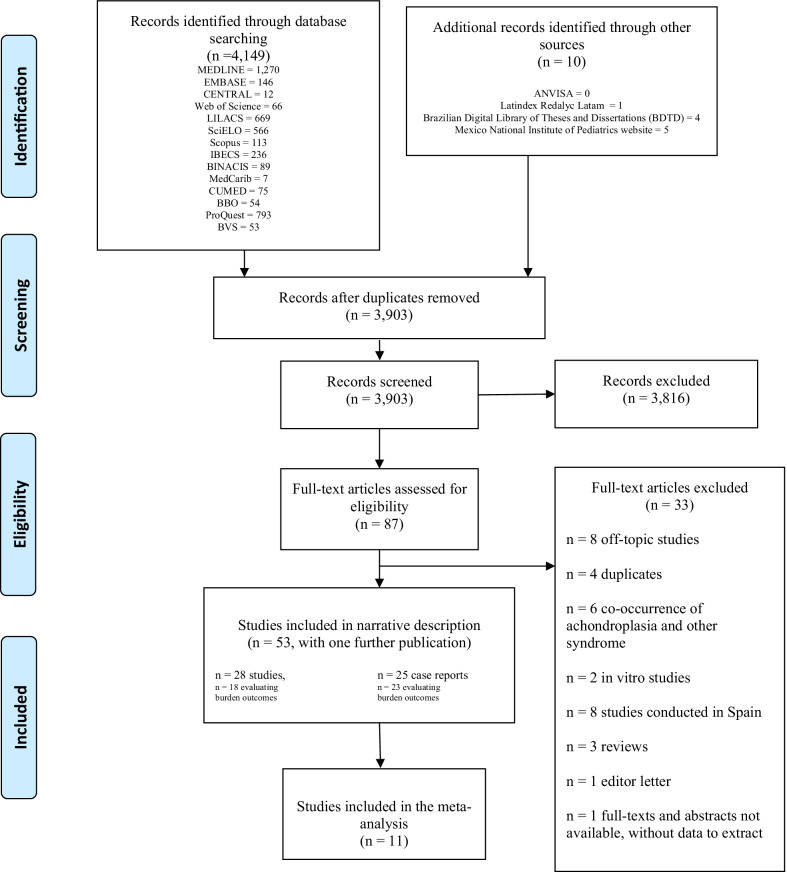
Search and selection of studies

Reviewers independently screened all titles and abstracts identified by the literature search using online software Covidence (https://www.covidence.org), obtained full-texts articles of all potentially relevant studies, and evaluated them against the eligibility criteria. Reviewers resolved disagreements by discussion or, if necessary, with third party adjudication. We also considered studies reported as abstracts; however, those that did not contain data to extract were excluded from the review. We recorded the selection process and completed a PRISMA (Preferred Reporting Items for Systematic Reviews and Meta-Analyses) flow diagram (Fig. 1).

### Data extraction

Reviewers independently extracted the following data using a pre-standardized data extraction form: (1) first author and year of publication; (2) country; (3) study design; (4) scenario; (5) age, gender, and body mass index (BMI); (6) number of patients; (7) eligibility criteria; and (8) patient-important and economic outcomes, if available. We avoided double counting of participants where there were multiple publications in the same population. If there was more than one published report of the same group of patients, the articles were analysed to verify whether they reported different outcomes. If they presented the same outcomes, we extracted the data from the most complete article. For studies that did not report BMI but provided height and weight we calculated this variable.

### Risk of bias assessment

For cohort and case–control studies, we planned to assess risk of bias with a modified version of the Ottawa-Newcastle instrument [18] that includes confidence in assessment of exposure and outcome; however, there was no included study classified as either cohort or case–control study.

For cross-sectional studies, we assessed risk of bias with the AXIS tool [19], though we excluded some domains not applied to our review. For case series and case reports, we used the single tool from the Joanna Briggs Institute (JBI) critical appraisal checklist for case reports [20]. However, in our view, the structure of the response options in both AXIS and JBI instruments leaves much to be desired. Therefore, we modified the response options to “definitely yes” (low risk of bias), “partially yes” (not all information needed available), “unclear” (no information to judge), and “definitely no” (high risk of bias), and applied it to our form for risk of bias in both cross-sectional and case series studies.

### Data synthesis and statistical analysis

We performed a systematic review of clinical studies with pooled analysis of proportions [21, 22], using the method of Stuart-Ord (inverse double arcsine square root).

Only case series and cross-sectional studies were considered for any quantitative analysis; case reports were excluded. We analyzed all outcomes as dichotomous variables with their respective confidence intervals (CI) of 95%. Since we expected that there were both clinical and methodological differences among the included studies, a random-effects model [23] was used to perform the pooled analysis of proportions. A statistically significant difference between two interventions required that their combined 95% CIs did not overlap [21, 22]. We calculated weighted mean and pooled measure of variability (standard deviation) for quality of life in the Cervan et al., 2008 [24] study as this study presented data of quality of life (QoL) for physical, psychological, social, environmental domains. The meta-analysis was performed with the StatsDirect software, version 2.8.0. (StatsDirect Ltd, Altrincham, Cheshire, UK).

Because of the very sparse data on this rare condition, when there was more than one report of the same type of burden outcome in the same study, we obtained the mean or median value from the subtype of outcome for the proportional meta-analysis to avoid selection bias. For example, on cardio-respiratory-metabolic disorders outcome, one study could report excessive snoring (number of events per number of total patients, 1/39), obesity (4/39), adenotonsillectomy (5/39), sleep disturbance (21/39), as well as sleep apnea (39/39). In this example, the median value used would be adenotonsillectomy (5/39).

### Subgroup and sensitivity analyses

We planned to perform subgroup analyses if there was a minimum of two studies in each category: (1) LATAM countries (e.g., Brazil versus Argentina); (2) adults versus children; however, there was an insufficient number of studies to allow for these assessments.

We performed a sensitivity analysis to explore causes of heterogeneity of the results, excluding studies according to study designs (i.e., case series versus cross-sectional studies).

### Heterogeneity assessment and publication bias

We investigated heterogeneity using the chi-square test and the I^2^ statistic [25]. An I^2^ value of 0–40%, 30–60%, 50–90% or 75–100% was interpreted as not important, moderate, substantial or considerable heterogeneity, respectively, and significance was assumed when I^2^ was > than 50% with a *p* < 0.1.

There was an insufficient number of studies (at least 10 or more) to allow for assessment of publication bias through visual inspection of funnel plots.

## Results

### Study selection

Our initial searches identified 4,149 citations. All were from electronic databases, except for ten studies identified through grey literature. After we removed duplicates from different databases, we retained 3,903 potentially relevant articles for further assessment. After reading titles and abstracts, 87 articles were retrieved as full text for further assessment. After screening the full texts, we included 53 clinical studies with one further publication. We excluded 32 studies after reviewing the full papers. The reasons for exclusion are listed in the PRISMA flow diagram (Fig. 1). The total number of included studies is 53 with one further publication, and from these 11 contributed to meta-analysis.

Eight of the included studies were published only as an abstract [26–33], and five studies as theses [34–38]. The remainder of the included studies (n = 40) was published as full-text articles [5, 6, 24, 39–74]. One study [75] was published as full-text with an additional publication in abstract format [76]. When information regarding risk of bias or other aspects of methods was unavailable, we attempted to contact study authors for additional information.

### Study characteristics

Tables 1 and 2 summarize the key characteristics from those studies that reported at least one patient-important or economic outcome. Regarding study design, four were case series [26, 34, 35, 73], 24 cross-sectional studies [5, 6, 24, 29, 36–38, 40, 45, 48, 50–54, 56, 58, 60, 61, 66, 69, 70, 72, 75], and 25 case reports [27, 28, 30–33, 39, 41–44, 46, 47, 49, 55, 57, 59, 62–65, 67, 68, 71, 74].

**Table 1:** LATAM ACH studies evaluating patient-important or economic burden outcomes not accountable for the meta-analysis

Author, year	South or Central America	LATAM country	Scenario	# of patients	Age, Mean (SD), y	Female, %	BMI, Mean (SD)	Exclusion criteria	Individual and/or population outcomes	Type of burden outcomes	Specify outcomes
*Case series studies*											
Cervantes [35]	Central America	Mexico	Rehabilitation service of the National Institute of Pediatrics	7	9	42.85	NR	Patients with previous lumbar spine instrumentation, patients with abdominal skin lesions, patients undergoing surgical procedures and abdominal exercises one month before the proposed date for starting physiotherapy	Individual	Spinal issues including stenosis, compression and associated pain	Patients received electrotherapy and spinal flexion exercises with an average of 18 therapy sessions for the correction of lumbar hyperlordosis
Dantas and Medeiros [26]	South America	Brazil	Medical Genetics Service at Alcides Carneiro University Hospital, Federal University of Campinas Grande	NR	NR	NR	NR	NR	Individual	Quality of life	QoL
										Others	Endocrinological data
										Nervous system disorders	Neurological problems
Cross-sectional studies											
Barbosa-Buck et al.^£^ [6]	South America	Argentina, Bolivia, Brazil, Chile, Colombia, Ecuador, Paraguay, Uruguay and Venezuela	Hospitals from nine South American countries	68	NR	NR	NR	NR	Individual	Premature mortality	Perinatal mortality
										Perinatal complications	Premature birth
Cervan et al.^£^ [24]	South America	Brazil	Small People Association of Brazil (AGPB)	21	32.70 (11.18)	61.90	33 (6.96)	NR	Individual	Quality of life	Physical; psychological; social; environmental
Gomez et al. [58]	South America	Colombia	Leather Design and Manufacturing Center	8	NR	NR	NR	NR	Population	Environmental burden	Adaptation of shoes, health economic impact
Lima [38]	South America	Brazil	Previously agreed location in the city of Sao Paulo	7	NR	14.28	NR	NR	Population	Psychological impact	Humiliation; stigma, recognition
										Socioeconomic burden	Labor market
Rocha and Wagner [69]	South America	Brazil	NR	8	NR	NR	NR	NR	Individual / Population	Orthopedic complications	Joint mobility
										Psychosocial complications	Perception of their psychosocial life
										Environmental burden	Challenges in physical activity
*Case report studies*											
Abrão et al. [39]	South America	Brazil	NR	1	29	Female	95.18	NR	Individual	Cardio-respiratory-metabolic disorders; and spinal issues including stenosis, compression and associated pain, and others	Use of a bronchofibroscope
Arlet et al. [41]	Central America	Mexico	Orthopedic Clinic, Benemérita Universidad Autónoma de Puebla	1	8	Female	NR	NR	Individual	Others	Horizontal overlap and crossbite
Benavides et al., 2018 [42]	South America	Colombia	NR	1	23	Female	36.76	NR	Individual	Pregnant patient Cardio-respiratory-metabolic disorders	Obesity, difficulty airway, difficulty ventilation and intubation, postoperative pulmonary complications, and cardiovascular complications such as risk of pulmonary hypertension crisis, acute heart failure, perioperative myocardial infarction during general anesthesia
										Spinal issues including stenosis, compression and associated pain	Neck instability and risk of spinal cord compression with neck, hyperextension, and difficult spinal and epidural puncture during regional anesthesia
Calderón et al. [43]	Central America	Cuba	University polyclinic “Ana Betancourt”	1	25	Female	NR	NR	NA^$^	NA^$^	NA^$^
Carbia et al.^¢^ [44]	South America	Argentina	Clinical Medical and Dermatology Divisions, Hospital "José María Ramos Mejía". Buenos Aires	1	83	Female	NR	NR	Individual	Others	Cicatricial metastasis as the presenting sign of squamous cell esophagus carcinoma
Carmen et al. [46]	South America	Chile	Obstetrics Service of the Ambato Regional Teaching Hospital	1	29	Female	27.54	NR	Individual	Pregnant patient Perinatal complications	Moderate anemia and cholecystitis
Carolina et al. [47]	South America	Chile	NR	1	1.10	Female	NR	NR	Individual	Spinal issues including stenosis, compression and associated pain Nervous system disorders	Aqueductal stenosis with symptomatic spinal cord compression
											Hydrocephalus
Castro [48]	South America	Brazil	Methodist University of Piracicaba (UNIMEP)	1	28	Female	28.47	NR	Individual / Population	Pain	Complaints of lower back and leg pain after a period of walking and low back pain caused by hyperlordosis
										Orthopedic complications	Varus foot
										Environmental burden	Difficulty getting on the bus because of the distance from the sidewalk to the step and the height of the steps
Eusebio and Vidal [55]	Central America	Dominican Republic	NR	1	4	Female	NR	NR	Individual	Spinal issues including stenosis, compression and associated pain Orthopedic complications	Lumbar hyperlordosis
											Bilateral femoral elongation surgery
Frade et al. [57]	South America	Brazil	University Hospital of Brasilia	1	0.1*	Female	18.5	NR	Individual	Perinatal complications	Needed positive pressure ventilation during birth, and during hospitalization, jaundice occurred
Galego et al. [27]	South America	Brazil	NR	1	32	Female	41.6	NR	Individual	Pregnant patient Perinatal complications	Emergency cesarean section due to umbilical cord prolapse
Hernández-Motiño et al. [59]	Central America	Mexico	Children's Hospital of Mexico Federico Gómez	1	5	Female	NR	NR	Individual	Cardio-respiratory-metabolic	Pulmonary arterial hypertension, apnea, and respiratory arrest, necessitating mechanical ventilation, making extubation impossible due to weakness of chest muscles
										Orthopedic complications	Limb functional limitation
										Spinal issues including stenosis, compression and associated pain	Compression of the cervicospinal canal
										Nervous system disorders	Delayed psychomotor development
										Others	Vesicostomy for neurogenic bladder
Jesus et al. [28]	South America	Brazil	Charitable Health Association of Northeast Paraná Norospar, Umuarama, PR	1	16	Female	37.5	NR	Individual	Pregnant patient Cardio-respiratory-metabolic disorders	Hypotension and dyspnea
										Pain	Pain at birth
Medina et al. [62]	South America	Paraguay	Pediatric Service Hospital Central, Institute of Social Security. Pediatric Intensive Care Unit	1	2.6	Female	NR	NR	Individual	Cardio-respiratory-metabolic disorders	Hospitalized for serious respiratory symptoms, admitted to pediatric ICU with assisted ventilation
										Spinal issues including stenosis, compression and associated pain	Medullary compression with decompressive surgery
Morais et al. [63]	South America	Brazil	Anesthesiology Department, Hospital Lifecenter, Belo Horizonte	1	47	Male	NR	NR	Individual	Spinal issues including stenosis, compression and associated pain	Thoracic kyphosis and severe lumbar lordosis, in addition to surgical scar on the lumbar region
										Others	Hemorrhoidectomy
Muratore and Viollaz [64]	South America	Argentina	Británico Hospital, Buenos Aires	1	21	Female	37.19	NR	Individual	Orthopedic complications	Bilateral femoral elongation surgery
Nascimento et al. [30]	South America	Brazil	Federal Hospital of Lagoa	1	51	Female	48.6	NR	Individual	Cardio-respiratory-metabolic disorders	Obesity and videolaparoscopic gastroplasty
Oliveira et al. [31]	South America	Brazil	Vera Cruz Hospital, Campinas / SP	1	33	Female	37.72	NR	Individual	Pregnant patient Pain others	Peritoneal plane pain at birth
										Others	Hypothyroidism and difficulty in epidural anesthesia for cesarean
Palmira et al. [65]	Central America	Cuba	Gyneco-obstetric University Hospital "Ana Betancourt de Mora" Camagüey	1	20	Female	NR	NR	Individual	Pregnant patient Cardio-respiratory-metabolic disorders	Obesity, bronchial asthma, and respiratory difficulty during pregnancy
										Spinal issues including stenosis, compression and associated pain	Lumbar hyperlordosis
										Others	Twin pregnancy of 34 weeks preventing the patient to walk, to stand up, and no tolerance of supine decubitus; 10 days of hospitalization after complications at birth with cesarean section
Pimentel and Figueiredo [67]	South America	Brazil	Professor Edgard Santos University Hospital Complex, Salvador	1	73	Male	46.82	NR	Individual	Others	Surgical treatment of colon adenocarcinoma, after surgery the patient was diagnosed with septic shock with an abdominal focus which required a new surgical approach, deep venous thrombosis, 30th day of hospitalization
Posada et al. [68]	South America	Colombia	NR	1	0.8*	Male	NR	NR	Individual	Pregnant patient Cardio-respiratory-metabolic disorders	Apnea
										Spinal issues including stenosis, compression and associated pain	Cervicomedullary compression, no cephalic control, chronic cervical myelopathy; spinal decompression
										Nervous system disorders	Seizures, irritability
										Orthopedic complications	Abolished tendon reflexes
Rudas et al. [71]	South America	Colombia	NR	1	29	Female	33.9	NR	Individual	Pregnant patient NA^$^	NA^$^
Tosato and Alves [32]	South America	Brazil	Hospital da Sagrada Família, Salvador, Bahia	1	22	Female	NR	NR	Individual	Pregnant patient NA^$^	NA^$^
Uemura et al. [74]	South America	Brazil	Ambulatory of the Specialization Course in Pediatric Dentistry of the Union of Dentists of the State of São Paulo (SOESP)	1	4	Female	NR	NR	Individual	Psychosocial complications	Does not accept his physical condition or maintain contact with other children
										Others	Anterior open bite
Werb et al. [33]	South America	Brazil	Getúlio Vargas State Hospital, Rio de Janeiro	1	79	Female	NR	NR	Individual	Others	Blood coagulation disorder with indication of suprapatellar amputation of lower limb

ACH: achondroplasia; ICU: intensive care unit; LATAM: Latin America; NR: not reported; NA: not applicable SD: standard deviation; BMI: body mass index

^¢^Hypochromic microcytic anemia (hematocrit 30) with hypoferremia and hypoproteinemia

^*^months

^#^number

^$^Case report study that did not evaluate any pre-defined burden outcomes

^£^Comparative cross-sectional studies

**Table 2:** LATAM ACH studies evaluating patient-important or economic burden outcomes accountable for the meta-analysis

Author, year	South or Central America	LATAM country	Scenario	# of patients	Age, Mean (SD), y	Female, %	BMI, Mean (SD)	Exclusion criteria	Individual and/or population outcomes	Type of burden outcomes	Specify outcomes
*Case series studies*											
Alves [34]	South America	Brazil	Neurosurgery Outpatient Clinic of Instituto Fernandes Figueira / Fiocruz	31	3.23	45.17	NR	Achondroplasia associated with other genetic diseases	Individual	Spinal issues including stenosis, compression and associated pain	Craniocervical decompression surgery after displaying signs of medullary suffering and alteration of tendon reflexes; surgical intervention (craniocervical decompression surgery and endoscopic third ventriculostomy)
										Nervous system disorders	Hydrocephalus as a neurosurgical complication; endoscopic third ventriculostomy was performed on one patient, successfully, that presented alterations of eye background with optic papilla edema, arterial hypertension, and convulsive crises; neurosurgical complications; accidental dural opening; need for reoperation to correct CSF fistula; surgical intervention (craniocervical decompression surgery and endoscopic third ventriculostomy)
										Others	Blood transfusion; length of ICU; length of hospitalization
Tello et al. [73]	South America	Argentina	Dr. Juan P. Garrahan Pediatric Hospital	76	NR	NR	NR	NR	Individual	Spinal issues including stenosis, compression and associated pain	Needed surgical procedure, given the poor spinal cord compliance to deformations; dorso-lumbar kyphosis; posterior fossa decompression; spinal cord liberation alone; anterior arthrodesis plus posterior instrumented arthrodesis; anterior arthrodesis, associated with fibular grafting followed by posterior simple arthrodesis; the same technique was employed on 2 other patients, followed by posterior arthrodesis instrumented with pedicular screws
										Nervous system disorders	Neurological problems
*Cross-sectional studies*											
Arita et al.^£^ [40]	South America	Brazil	Heliopolis Hospital, Sao Paulo	11	40.27 (8.09)	45.45	33.75 (7.94)	NR	Individual	Ear, nose, throat and speech disorders	Mean number of teeth; bone fracture; mean cortical width; Klemetti index
										Spinal issues including stenosis, compression and associated pain	Normal density; osteopenia/osteoporosis; mandibular cortical erosion in all sample
Ceroni et al. [5]	South America	Brazil	Medical School of the University of Sao Paulo (HC-FMUSP)	39	10.2	NR	NR	Patients who did not present a molecular study	Individual	Ear, nose, throat and speech disorders	Motor developmental delay; speech delay; recurrent infection of the middle ear; persistent middle ear fluid; hearing loss; ventilation tube insertion
										Cardio-respiratory-metabolic disorders	Sleep disturbance; sleep apnea; adenotonsillectomy; excessive snoring; obesity
										Pain	Lower limb pain; excessive bowing of limbs
										Depression, anxiety, bullying, isolation, somatization, etc	Depression
										Nervous system disorders	Hydrocephalus; epilepsy
										Perinatal complications	Perinatal intercurrence; premature, presenting respiratory distress, pathological jaundice and deglutition disturbance; required three months of hospitalization
										Other	Bilateral Wilms’ tumor
Escobar [37]	Central America	Mexico	National Institute of Pediatrics of the Ministry of Health	87	NR	39	NR	Patients with other achondrodysplasias	Individual	Nervous system disorders	Hydrocephalus; paresthesias; paresias; hypotonia
										Spinal issues including stenosis, compression and associated pain	Spinal compression; surgical treatment, with posterior laminectomy being the procedure performed
										Cardio-respiratory-metabolic disorders	Apnea followed by death; neuromuscular blockers
										Ear, nose, throat and speech disorders	Recurrent otitis media; required surgical treatment (placement of ventilation tubes)
										Orthopedic complications	Orthopedic complication; corrective treatment; lengthening treatment
										All-cause mortality	Sudden death
										Others	Eye complications
Fano and Lejarraga [56]	South America	Argentina	Consultancy services for the growth and development of the National Hospital of Pediatrics "Prof. Dr. J. P. Garrahan"	96	3.07	47.91	NR	NR	Individual	Ear, nose, throat and speech disorders	Relapsing otitis media; any degree of hearing loss; delay in speech development; hipotonia
										Cardio-respiratory-metabolic disorders	Recurrent bronchitis; pneumonia; snoring during sleep; surgical requirement for presenting signs of cervicomedullary compression plus coexistence with severe respiratory illness; obesity
										Spinal issues including stenosis, compression and associated pain	Rigid kyphosis; spinal compression requiring laminectomy
										Nervous system disorders	Any neurological complications; surgical requirement for presenting signs of cervicomedullary compression (decompressive surgery of foramen magnum); surgical requirement for presenting signs of cervicomedullary compression plus coexistence with severe respiratory illness; delay in motor development; hydrocephalus that required ventriculoperitoneal shunt
										Premature mortality	Patients died of respiratory failure
										Others	Sustained hypoxemia requiring home oxygen supply due to sequelae of lung disease after infection
Junior [29]	South America	Brazil	Rehabilitation hospital in Minas Gerais	24	NR	54.16	NR	NR	Individual	Nervous system disorders	Neurological manifestations; low thoracolumbar comprehensive myeloradiculopathy
Medeiros et al., Medeiros et al. [75, 76]	South America	Brazil	Medical Genetics outpatient clinic at Hospital Universitário Alcides Carneiro (HUAC) from the Federal University of Campinas Grande	8	NR	37.5	NR	NR	Individual / Population	Ear, nose, throat and speech disorders	Hypertrophy of adenoids; snoring; tonsillectomy; thickening of the tympanic membrane; hearing loss; recurrent otitis media
										Cardio-respiratory-metabolic disorders	Apnea index moderately highly increased; desaturations during sleep, but not accompanied by electrocardiographic change
										Environmental burden	Challenges in physical activity
Petitto and Baumotte [66]	South America	Brazil	Participants' home	5	25	NR	33.66	NR	Individual	Psychosocial complications (i.e., impact of the disease on patient)	Body image
Rodriguez-Gomez et al. [70]	Central America	Puerto Rico	Residents of Puerto Rico	22	39.6	68.2	NR	NR	Individual	Depression, anxiety, bullying, isolation, somatization, etc	Mild to severe depressive symptoms; mild to severe symptoms associated to anxiety; mild to severe symptoms associated with hopelessness; mild to severe symptoms in at least one of the sub-scales in Derogatis Symptom Checklist-90-Revisited (SCL-90-R) particularly the obsessive-compulsive, paranoid and depressive subscales; mild somatization; mild interpersonal sensitivity; mild hostily; mild to severe paranoid ideation; mild to moderate psychoticism
										Cardio-respiratory-metabolic disorders	At least one complication (hypertension, diabetes, rheumatoid arthritis, asthma, scoliosis, thyroid problems, neuropathy, psoriasis, gastritis and/or sleep apnea)
										Nervous system disorders	
										Ear, nose, throat and speech disorders	
										Spinal issues including stenosis, compression and associated pain	
										Others	Diabetes, rheumatoid arthritis, thyroid problems, neuropathy, psoriasis, and gastritis
Sanchez et al. [72]	South America	Venezuela	Ruiz y Páez Hospital in Ciudad Bolívar	10	NR	NR	NR	NR	Individual	Premature mortality	Died at 10 months of age due to respiratory complications
										Nervous system disorders	Convulsions and mental retardation
										Others	Acanthosis Nigricans

ACH: achondroplasia; ICU: intensive care unit; LATAM: Latin America; NR: not reported; NA: not applicable SD: standard deviation; BMI: body mass index

^#^Number

^£^Comparative cross-sectional studies

Twenty-four of the included studies were conducted in Brazil [5, 24, 26–34, 36, 38–40, 45, 49, 57, 63, 66, 67, 69, 74, 75], nine in Argentina [44, 50–54, 56, 64, 73], five in Colombia [42, 48, 58, 68, 71], four in Mexico [35, 37, 41, 59], three in Chile [46, 47, 61], three in Cuba [43, 60, 65], one each in Dominican Republic [55], in Paraguay [62], in Venezuela [72], and in Puerto Rico [70]. Only one article [6] was a multicenter cross-sectional study, which involved nine countries (i.e., Argentina, Bolivia, Brazil, Chile, Colombia, Ecuador, Paraguay, Uruguay, and Venezuela) [6]. Sample sizes from these studies ranged from four [61] to 357 patients [53]. Study participants ranged in age, from a mean age of 3.07 [56] to 40.24 [40] years (Tables 1 and 2).

The type of burden outcome most frequently reported among the cross-sectional and case series studies was nervous system disorders (28.66%, n = 8) [5, 29, 34, 37, 56, 70, 72, 73], followed by spinal issues including stenosis, compression and associated pain (25.00%, n = 7) [34, 35, 37, 40, 56, 70, 73], and then ear, nose, throat and speech disorders (21.42%, n = 6) [5, 37, 40, 56, 70, 75]. The majority of the cross-sectional, case series, and case reports studies (86.36%, n = 38) reported only on patient-important outcomes (Tables 1 and 2).

Additional file 2: Table S2 describes study characteristics related to LATAM countries only from those that reported other than patient-important or economic outcome. Ten studies [36, 45, 48, 50–54, 60, 61] evaluated in addition to burden outcomes, such factors as mutations in the fibroblast growth factor receptor 3 gene [48, 61]; growth velocity [52, 54]; and body proportions references [53].

Additional file 3: Table S3 describes the burden outcomes on 25 LATAM case reports studies. With regards the case reports studies, the majority (68.0%, n = 17) [27, 28, 30, 39, 42, 44, 46, 47, 49, 55, 57, 59, 62–65, 68] assessed some physical comorbidities such as apnea [59, 68], lower back and leg pain [49], and obesity [65]. Ten case report studies evaluated other outcomes such as hemorrhoidectomy [63] and vesicostomy for neurogenic bladder [59]. Only one study [49] reported on environmental burden (i.e., difficulty getting on the bus because of the distance from the sidewalk to the step and the height of the steps).

### Risk of bias assessment

**Fig. 2:**
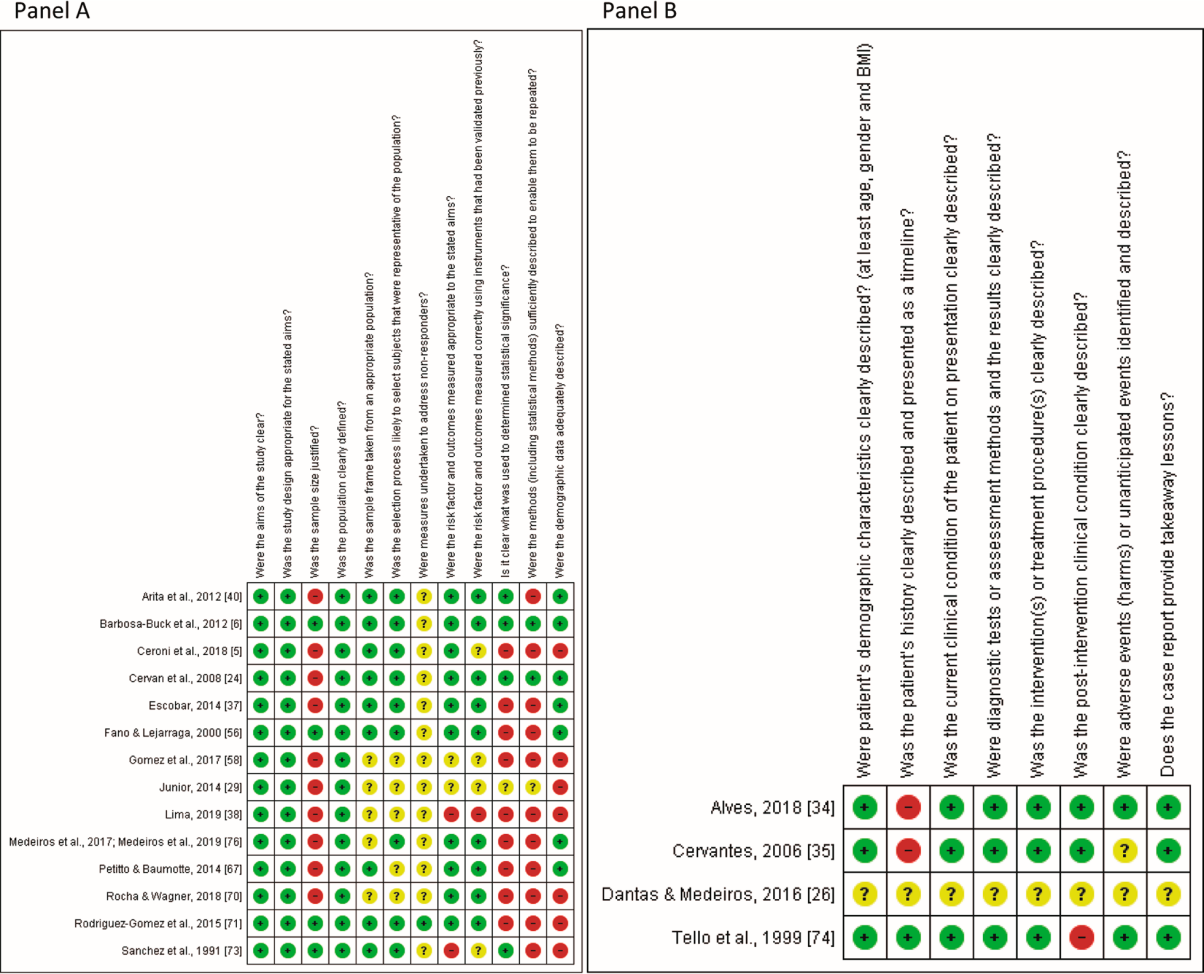
Risk of bias assessment of the included studies. **a** Cross-sectional studies. **b** Case series studies

Figure 2 and Additional files 4, 5: Tables S4 and S5 describe the risk of bias assessment. In the cross-sectional studies (Fig. 2, panel A), at least one of the following domains of sample size, statistical significance, statistics methods, or demographic data were rated as “high risk of bias” in 13 studies (54.16%) [5, 24, 29, 37, 38, 40, 56, 58, 66, 69, 70, 72, 75]. In the case series studies (Fig. 2, panel B), only two domains (i.e., clear description of both patient’s history and post-intervention clinical condition) were rated as “high risk of bias” in three studies (75.00%) [34, 35, 73].

### Outcomes

The results were pooled from studies that reported available data. Therefore, out of 54 included studies [5, 6, 24, 26–76], only 11 [5, 29, 34, 37, 40, 56, 66, 70, 72, 73, 75, 76] were used for the quantitative analysis described below as they presented available data (Table 2).

#### Mortality

**Fig. 3:**
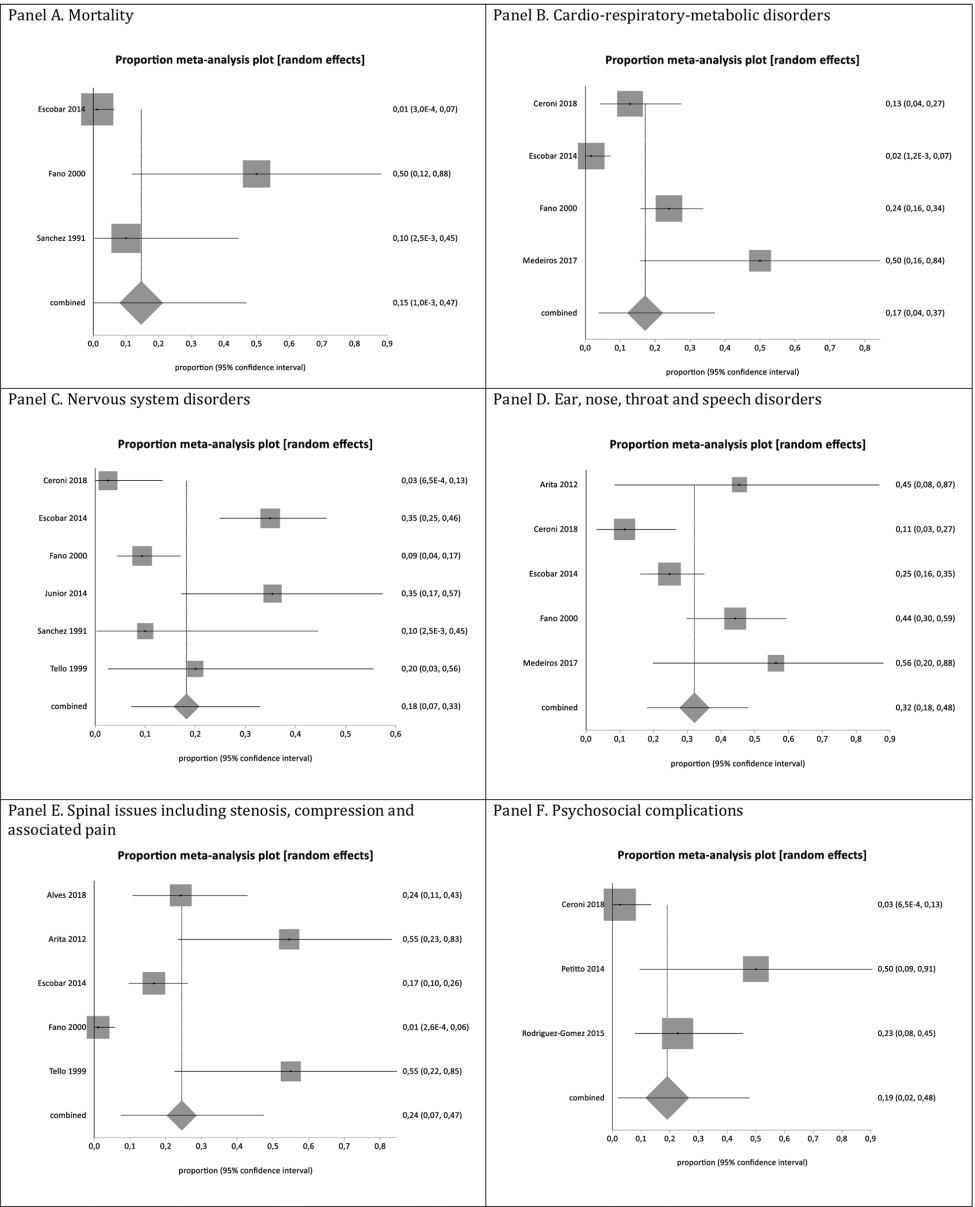
Pooled analysis of proportions for burden outcomes in LATAM ACH patients. **a** Mortality. **b** Cardio-respiratory-metabolic disorders. **c** Nervous system disorders. **d** Ear, nose, throat and speech disorders. **e** Spinal issues including stenosis, compression and associated pain. **f** Psychosocial complications. **g** Others

The pooled proportion for mortality (i.e., sudden death [37] and death due to respiratory complications [56, 72]) was 15% [95% CI 1.0E−3 to 0.47; I2 = 82.9%, *p* = 0.0029] from three studies [37, 56, 72] with a total of 99 patients (Fig. 3). There was significant statistical heterogeneity in the analyses.

#### Cardio-respiratory-metabolic disorders

The pooled proportion for cardio-respiratory-metabolic disorders was 17% [95% CI 0.04 to 0.37; I2 = 90.3%, *p* < 0.0001] from four studies [5, 37, 56, 75, 76] with a total of 230 patients (Fig. 3). There was significant statistical heterogeneity in the analyses. The outcomes used to calculate the mean or median of the cardio-respiratory-metabolic disorders among the studies included in the analysis were: adenotonsillectomy [5]; apnea followed by death [37]; pneumonia [56]; apnea index slightly and moderately increased [75]; desaturations during sleep [75]; and apnea [75]. There was no outcome directly related to cardiac to be included in this category.

#### Nervous system disorders

**Fig. 4:**
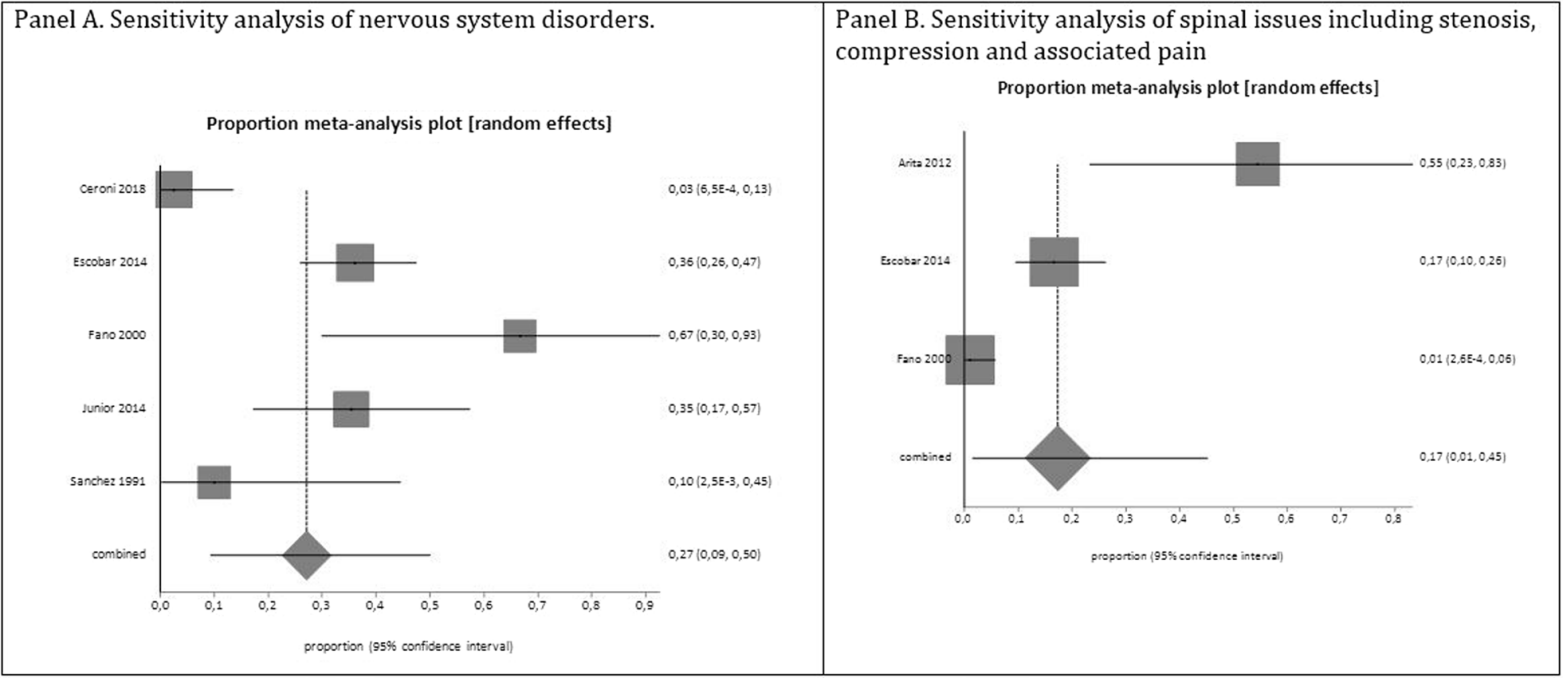
Sensitivity analysis excluding case series studies from the cross-sectional studies for burden outcomes in LATAM ACH patients. Panel A: Nervous system disorders. Panel B: Spinal issues including stenosis, compression and associated pain.

The pooled proportion for nervous system disorders was 18% [95% CI 0.07 to 0.33; I2 = 84.6%, *p* < 0.0001] from six studies [5, 29, 37, 56, 72, 73] with a total of 262 patients (Fig. 3). There was significant statistical heterogeneity in the analyses. A sensitivity analysis excluding case series studies from the cross-sectional studies yielded results that were consistent with the primary analysis of 27% [95% CI 0.09 to 0,50; I2 = 87.2%, *p* < 0.0001] from five studies [5, 29, 37, 56, 72] with a total of 165 patients (Fig. 4). There was no statistically significant difference between the primary analysis (i.e., all the studies) and the sensitivity analysis (i.e., only cross-sectional studies), as their CIs overlapped. The outcomes used to calculate the mean or median of the nervous system disorders among the studies included in the analysis were: hydrocephalus [5, 37]; convulsive crises [72]; epilepsy [5]; paresthesias and paresias [37]; hypotonia [37]; neurological manifestations [56]; decompressive surgery of foramen magnum [56]; mental retardation [72]; and neurological problems [73].

#### Ear, nose, throat and speech disorders

The pooled proportion for ear, nose, throat and speech disorders was 32% [95% CI 0.18 to 0.48; I2 = 73.4%, *p* = 0.0046] from five studies [5, 37, 40, 56, 75, 76] with a total of 183 patients (Fig. 3). There was significant statistical heterogeneity in the analyses. The outcomes used to calculate the mean or median of the ear, nose, throat and speech disorders among the studies included in the analysis were: hearing loss [5, 56, 75]; recurrent otitis media [37, 56, 75]; required surgical treatment (i.e., placement of ventilation tubes) [37]; delay in speech development [56]; hypotonia [56]; hypertrophy of adenoids [75]; snoring; tonsillectomy; and thickening of the tympanic membrane [75].

#### Spinal issues including stenosis, compression and associated pain

The pooled proportion for spinal issues including stenosis, compression and associated pain was 24% [95% CI 0.07 to 0.47; I2 = 91.3%, *p* < 0.0001] from five studies [34, 37, 40, 56, 73] with a total of 235 patients (Fig. 3). There was significant statistical heterogeneity in the analyses. A sensitivity analysis excluding case series studies from the cross-sectional studies yielded results that were consistent with the primary analysis of 17% [95% CI 0.01 to 0.45; I2 = 93.4%, *p* < 0.0001] from three studies [37, 40, 56] with a total of 194 patients (Fig. 4). There was no statistically significant difference between the primary analysis (i.e., all the studies) and the sensitivity analysis (i.e., only cross-sectional studies), as their CIs overlapped. The outcomes used to calculate the mean or median of the spinal disorders among the studies included in the analysis were: osteopenia or osteoporosis [40]; posterior laminectomy [37]; craniocervical compression [34, 37, 56, 73]; spinal compression requiring laminectomy [56]; spinal cord liberation alone [73]; anterior arthrodesis plus posterior instrumented arthrodesis [73]; anterior arthrodesis, associated with fibular grafting followed by posterior simple arthrodesis [73]; posterior arthrodesis instrumented with pedicular screws [73].

#### Psychosocial disorders

The pooled proportion for psychosocial complications was 19% [95% CI 0.02 to 0.48; I2 = 80.8%, *p* = 0.0054] from three studies [5, 66, 70] with a total of 66 patients (Fig. 3). There was significant statistical heterogeneity in the analyses. The outcomes used to calculate the mean or median of the psychosocial disorders among the studies included in the analysis were: depression [5], perception of their body image [66], and mild somatization [70].

### Descriptive analysis

Four studies [38, 58, 69, 75, 76] reported on economic burden outcomes. Gomez et al., 2017 [58] reported on the adaptation of shoes for the ACH patients and the costs associated with the anthropometric and baropodometric analyses of the foot. This study addressed the design of a footwear system that fulfills form, function and usage of eight persons with ACH patients. The most relevant information was that footwear should have a low heel (about 2 cm) as there is a greater risk of falling due to the instability associated with wearing higher heels (7 1⁄2 cm and above), considering the lower center of gravity for ACH patients; however, patients want to have comfort and elegant heels and shoes (Table 1).

The Lima, 2019 [38] study sought to identify the consequences of stigmatization on social life, including work. The results indicate that people with ACH experience humiliation and disrespect due to associations made with the stereotype built about them. The authors found this stereotype is commonly used by comedians for entertainment purposes (Table 1).

Medeiros et al., 2017; Medeiros et al., 2019 [75, 76] and Rocha & Wagner, 2018 [69] describe the challenges associated with physical activities (Table 1). Patients reported that while physical activities can be difficult to perform [75, 76], though the regular practice of physical activity improves their self-esteem and confidence which in turn contributes to their sense of social inclusion [69].

None of the included studies reported on the following patient-important outcomes: suicide attempts, and suicide rates; impact of the disease on caregivers, such as health-related quality of life, activities of daily living, work productivity, education, employment, social, and so forth; and social adaptation challenges. Furthermore, none of the included studies reported on the following economic burden outcomes: limitation of physical access to transportation modalities; and adaptation to standard transport equipment.

## Discussion

### Main findings

Based on pooled data from 11 clinical studies [5, 29, 34, 37, 40, 56, 66, 70, 72, 73, 75, 76] with 409 participants, we found evidence of the impact of ACH on affected individuals in LATAM. Case-series and cross-sectional studies provide pooled proportions of burden ranging from 15% (mortality) to 32% (for ear, nose, throat and speech disorders outcomes).

We have now applied a methodology [21, 22] to evaluate the proportions of clinical burden outcomes in the LATAM ACH population. The proportions of pooled case series and cross-sectional studies were consistent with results from only pooled cross-sectional studies in the outcomes of nervous system disorders and spinal issues including stenosis, compression and associated pain, meaning that the assumed proportions lie in a high probability of a true value. To the best of our knowledge, this is the first study to clearly demonstrate the burden of LATAM ACH patients, an observation that should be taken into account in regional health policy debates regarding management of ACH disease. Of note, while limb lengthening procedures are frequently performed on ACH patients in LATAM, we were not able to find any data on these procedures in the included studies.

### Strengths and limitations

Strengths of our review include a comprehensive search; assessment of eligibility, risk of bias and data abstraction independently and in duplicate; and an assessment of risk of bias that included a sensitivity analysis addressing homogeneity of study designs.

The primary limitation of our study is related to the rare disease nature of ACH. The population available to study was limited and the study designs presented some flaws.

Another limitation is that our analysis demonstrates a significant heterogeneity (I^2^) in all studied clinical burden outcomes. Explanations for this heterogeneity could be both clinical and methodological diversities. The studies differed considerably in their mean age of patient selection, study designs (i.e., case series, cross-sectional, and case reports), and type of burden outcomes (e.g., nervous system disorders, one study could report hydrocephalus, while another reports epilepsy).

Furthermore, out of the 53 clinical studies we were only able to include data in the meta-analysis from 11 of them (20.4%). The majority of the studies were difficult to decipher, and they did not provide all burden pre-defined outcomes.

A further limitation was the insufficient number of studies, which prevented completing statistical analyses that had initially been planned. We were unable to assess publication bias because there were less than 10 eligible studies addressing the same outcome in a meta-analysis. Subgroup analyses on LATAM countries (e.g., Brazil versus Argentina), and adults versus children were not possible since minimal criteria were not met (i.e., at least four studies available, with at least two in each sub-group).

A sensitivity analysis pooling all included studies (i.e., case series and cross-sectional studies) compared with pooling only the cross-sectional studies was only possible for nervous system disorders and spinal issues outcomes. No difference was found in the proportion of overlap CIs between both analyses.

### Relation to prior research

Unfortunately, there is a dearth of information on LATAM patients in both non-English and English-language medical literature on ACH. While we found a 15% mortality rate in our review, with a wide-ranging prevalence from 0.1 to 47%, a multicenter study of mortality in ACH [77] that studied 855 USA patients presented an overall mortality rate of 99% per 1,000 person years, with an absolute number of deaths of 12 patients (n = 5, toddlers; n = 2, young children; and n = 5, young adulthood). The authors of the clinical study also found that the infant mortality rate was 3.2/1,000 person years. Although only three studies [37, 56, 72] contributed to our data on mortality, only one of them [56] reported the mean age of the population as 3.07 years old. Therefore, in our systematic review, 33.33% of the mortality data is contributed from predominantly children with a mean age of three years. Another two studies have estimated the maximal risk of deaths in the first year of life as 7.5% [2, 9]; this high percentage may also be explained due to the absence of special care and surgical intervention.

Furthermore, an additional two studies [78, 79] identified in the literature corroborate with our findings showing that motor delays are common in the ACH population. In our review, we found a prevalence of nervous system disorders, which encompassed motor delay, of 26% with a reasonable CI ranging from 12 to 44%.

A high prevalence of ear, nose, and throat disorders (32%) and spinal issues (26%) were found in our review which is consistent with other studies indicating that middle ear dysfunction [80] and spinal stenosis [81] are highly common in both children and adults with ACH.

## Conclusions

LATAM ACH patients presented a high prevalence of clinical complications, although the possibility of residual confounding due to lack of adequate reports in this population and high heterogeneity in the analysis cannot be ruled out. This study also highlights the need to address well-conducted clinical studies on ACH, and to alert the public health authorities. Future observational studies should have standardized outcomes measures such as mortality, physical comorbidities, humanistic outcomes, and socioeconomic and environmental burden outcomes.

## Supplementary Information

**Additional file 1**. Search strategy.

**Additional file 2**. Ten LATAM ACH included studies evaluating other than patient-important or economic burden outcomes.

**Additional file 3**. Reported burden outcomes on 25 LATAM ACH case reports studies.

**Additional file 4**. Risk of bias for cross-sectional studies.

**Additional file 5**. Risk of bias for case series studies.

## Data Availability

All data generated or analysed during this study are included in this published article and its supplementary information files.

## Abbreviations

ACH: Achondroplasia
LATAM: Latin American
ECLAMC: Latin American Collaborative Study of Congenital Malformations
PRISMA: Preferred Reporting Items for Systematic Review and Meta-Analysis
MOOSE: Meta-analysis of Observational Studies in Epidemiology
PROSPERO: International Prospective Register of Systematic Reviews
BPI-SF: Brief Pain Inventory-Short Form
QoLiSSY: Questionnaire, the Quality of Life Short Stature Youth
PedsQL: Questionnaire, and the Pediatric Quality of Life Inventory
WPAI-SHP: Work and Productivity and Activity Impairment
MeSH: Medical Subject Headings
MEDLINE: Medical Literature Analysis and Retrieval System Online
EMBASE: Excerpta Medica Database
CENTRAL: Cochrane Central Register of Controlled Trials
LILACS: Latin American and Caribbean Health Sciences Literature
SciELO: Scientific Electronic Library Online
IBECS: Spanish Bibliographic Index of the Health Sciences
BINACIS: National Bibliography in Health Sciences Argentina
MedCarib: Caribbean Health Sciences Literature
CUMED: National Medical Sciences Information Center of Cuba
BBO: Brazilian Bibliography of Dentistry
ANVISA: National Health Surveillance Agency
BDTD: Brazilian Digital Library of Theses and Dissertations
BMI: Body mass index
JBI: Joanna Briggs Institute
QoL: Quality of life
CI: Confidence Interval
