# Supplementary material for: Impact of achondroplasia on Latin American patients: a systematic review and meta-analysis of observational studies
Source: Orphanet J Rare Dis. 2022 Jan 4;17:4. doi: 10.1186/s13023-021-02142-3 (PMC8728937; doi:10.1186/s13023-021-02142-3)
Supplement: Supplementary file 1 — Additional file 1. Search strategy. [file 13023_2021_2142_MOESM1_ESM.docx]

**Supplementary Table 1.** Search strategy.

| ((Achondroplasia OR Achondroplasias OR achondroplastic OR skeletal dysplasia OR skeletal dysplasias OR short stature OR dwarfism OR dwarf OR osteochondrodysplasias OR Osteochondrodysplasia OR Hyperostosis Corticalis Generalisata OR Hyperphosphatasemia Tarda OR Sost-Related Sclerosing Bone Dysplasia OR Sost Sclerosing Bone Dysplasia OR Autosomal Recessive Endosteal Hyperostosis OR Van Buchem Disease OR Melnick-Needles Syndrome OR Melnick Needles Syndrome OR Osteodysplasty of Melnick and Needles OR Melnick-Needles Osteodysplasty OR Melnick Needles Osteodysplasty OR Multiple Epiphyseal Dysplasia OR Myotonic Chondrodystrophy OR Schwartz-Jampel-Aberfeld Syndrome OR Chondrodystrophic Myotonia OR Schwartz Jampel Aberfeld syndrome OR Spondylo-Epimetaphyseal OR Dysplasia With Myotonia OR Schwartz-Jampel Syndrome OR Schwartz Jampel Syndrome OR Myotonic Myopathy, Dwarfism, Chondrodystrophy, And Ocular And Facial Abnormalities OR SJA Syndrome OR Spondyloepiphyseal Dysplasia OR X-Linked Spondyloepiphyseal Dysplasia Tarda OR X Linked Spondyloepiphyseal Dysplasia Tarda OR Late-Onset Spondyloepiphyseal Dysplasia OR Late Onset Spondyloepiphyseal Dysplasia OR X-Linked SED OR X Linked SED OR X-Linked SEDT OR X Linked SEDT OR Late Spondyloepiphyseal Dysplasia OR SED Tarda OR Type 1 Schwartz-Jampel Syndrome OR Type 1 Schwartz Jampel Syndrome OR Dyschondroplasias OR Dyschondroplasia OR Skeleton-Skin-Brain Syndrome OR Skeleton Skin Brain Syndrome OR Skeleton-Skin-Brain Syndromes OR Severe Achondroplasia with Developmental Delay and Acanthosis Nigricans OR SADDAN OR SADDANs OR SADDAN Dysplasia OR SADDAN Dysplasias OR Hypochondroplasia OR Pseudoachondroplasia) AND (Latin America OR South America OR Latin America country OR South America country OR OR Latin America countries OR South America countries OR Argentina OR Bolivia OR Brazil OR Chile OR Colombia OR Ecuador OR French Guiana OR Guyana OR Paraguay OR Peru OR Suriname OR Uruguay OR Venezuela OR LATAM country OR LATAM countries OR LATAM)) AND (human OR humans) |
| --- |
