# Supplementary material for: Impact of achondroplasia on Latin American patients: a systematic review and meta-analysis of observational studies
Source: Orphanet J Rare Dis. 2022 Jan 4;17:4. doi: 10.1186/s13023-021-02142-3 (PMC8728937; doi:10.1186/s13023-021-02142-3)
Supplement: Supplementary file 2 — Additional file 2. Ten LATAM ACH included studies evaluating other than patient-important or economic burden outcomes. [file 13023_2021_2142_MOESM2_ESM.docx]

**Supplementary Table 2.** Ten LATAM ACH included studies evaluating **other than** patient-important or economic burden outcomes.

| Author, year | LATAM country | Scenario | # of participants | Age, Mean (SD), y | Female, % | BMI | Outcomes evaluated other than those established in this review |
| --- | --- | --- | --- | --- | --- | --- | --- |
| Cardoso et al., 2012^£^ [45] | Brazil | NR | 14 | NR | 57.14 | NR | Position of the cranial base, maxila, and mandible. |
| Castro et al., 2010 [48] | Colombia | Small Giants Association of Colombia. | 20 | 0.1* to 53 | 55 | NR | Mutations in the Fibroblast Growth Factor Receptor 3 gene (FGFR3). |
| Costa, 2010 [36] | Brazil | City of Belem, Para. | 11 | 25 (5.87) | 0 | 30.88 (6.37) | Anthropometric, dermatoglyphic characteristics and assessment of basic physical qualities. |
| Del Pino et al., 2010^£^ [50] | Argentina | Growth Clinic at Hospital Garrahan. | 244 | NR | 52.04 | NR | Growth references for height, weight, and head. |
| Del Pino & Fano, 2013^£^ [51] | Argentina | Hospital Growth and Development Service Garrahan. | 269 | NR | 52.04 | NR | Establish reference values for BMI. |
| Del Pino et al., 2018a^£^ [52] | Argentina | Growth Clinic at Hospital Garrahan. | 23 | 9.75 (1.15) | 65.21 | NR | Growth velocity and biological variables during puberty. |
| Del Pino et al., 2018b^£^ [53] | Argentina | Growth Clinic at Hospital Garrahan. | 359 | NR | 50 | NR | Leg length, sitting height, and body proportions references. |
| Del Pino et al., 2019^£^ [54] | Argentina | Growth Clinic at Hospital Garrahan. | 84 | NR | 48.8 | NR | Height growth velocity during infancy and childhood. |
| Lotti et al., 1998 [61] | Cuba | 36 Gynecological and obstetric hospitals in 13 Cuban provinces. | 23 | NR | NR | NR | Congenital malformations at birth. |
| Mancilla et al., 2003 [62] | Chile | Faculty of medicine of the Pontificia Universidad Católica de Chile. | 4 | NR | NR | NR | Mutations in the Fibroblast Growth Factor Receptor 3 gene (FGFR3). |

ACH: achondroplasia; LATAM: Latin America; NR: not reported; SD: standard deviation; BMI: body mass index.

#number.

*months.

^£^Comparative cross-sectional study.
