# Supplementary material for: Impact of achondroplasia on Latin American patients: a systematic review and meta-analysis of observational studies
Source: Orphanet J Rare Dis. 2022 Jan 4;17:4. doi: 10.1186/s13023-021-02142-3 (PMC8728937; doi:10.1186/s13023-021-02142-3)
Supplement: Supplementary file 3 — Additional file 3. Reported burden outcomes on 25 LATAM ACH case reports studies. [file 13023_2021_2142_MOESM3_ESM.docx]

**Additional File 3.** Reported burden outcomes on 25 LATAM ACH case reports studies.

|  | **Individual level** | **Mortality** | **Physical** | **Psychosocial** | **Others** | **Population level** | **Socioeconomic** | **Environmental** | **Health economic impact** |
| --- | --- | --- | --- | --- | --- | --- | --- | --- | --- |
| **Abrão et al., 2009 [39]** |  |  | X |  | X^$^ |  |  |  |  |
| **Arlet et al., 2016 [41]** |  |  |  |  | X^¢^ |  |  |  |  |
| **Benavides et al., 2018 [42]** |  |  | X |  |  |  |  |  |  |
| **Calderón et al., 2010**^£^ **[43]** |  | NA^£^ | | | |  | NA^£^ | | |
| **Carbia et al., 2003 [44]** |  |  | X |  | X^¥^ |  |  |  |  |
| **Carmen et al., 2019 [46]** |  |  | X |  |  |  |  |  |  |
| **Carolina et al., 2010 [47]** |  |  | X |  |  |  |  |  |  |
| **Castro, 2012 [48]** |  |  | X |  |  |  |  | X |  |
| **Eusebio & Vidal, 1991 [55]** |  |  | X |  |  |  |  |  |  |
| **Frade et al., 2012 [57]** |  |  | X |  |  |  |  |  |  |
| **Galego et al., 2018 [27]** |  |  | X |  |  |  |  |  |  |
| **Hernández-Motiño et al., 2012 [59]** |  |  | X |  | X^€^ |  |  |  |  |
| **Jesus et al., 2017 [28]** |  |  | X |  |  |  |  |  |  |
| **Medina et al., 2008 [62]** |  |  | X |  |  |  |  |  |  |
| **Morais et al., 2006 [63]** |  |  | X |  | X^₠^ |  |  |  |  |
| **Muratore et al., 2010 [64]** |  |  | X |  |  |  |  |  |  |
| **Nascimento et al., 2015 [30]** |  |  | X |  |  |  |  |  |  |
| **Oliveira et al., 2015 [31]** |  |  |  |  | X^α^ |  |  |  |  |
| **Palmira et al., 2012 [65]** |  |  | X |  | X^β^ |  |  |  |  |
| **Pimentel & Figueiredo, 2019 [67]** |  |  |  |  | X^θ^ |  |  |  |  |
| **Posada et al., 2005 [68]** |  |  | X |  |  |  |  |  |  |
| **Rudas et al., 2012 [71]** |  | NA^£^ | | | |  |  | | |
| **Tosato & Alves, 2017 [32]** |  | NA^£^ | | | |  |  | | |
| **Uemura et al., 2002 [74]** |  |  |  | X | X^ω^ |  |  |  |  |
| **Werb et al., 2016 [33]** |  |  |  |  | X^γ^ |  |  |  |  |

^$^Difficulty in tracheal intubation awake under direct laryngoscopy, requiring the use of a bronchofibroscope.

^¢^Horizontal overlap and crossbite.

^£^Case report study that did not evaluate any pre-defined burden outcomes.

^¥^Cicatricial metastasis as the presenting sign of squamous cell esophagus carcinoma.

^€^Vesicostomy for neurogenic bladder.

^₠^Hemorrhoidectomy.

^α^Hypothyroidism and difficulty in epidural anesthesia for cesarean.

^β^Twin pregnancy of 34 weeks avoiding the patient to walk, to stand up, and no tolerance of supine decubitus; 10 days of hospitalization after complications at birth with cesarean section.

^θ^Surgical treatment of colon adenocarcinoma, after surgery the patient was diagnosed with septic shock with an abdominal focus which required a new surgical approach, deep venous thrombosis, 30th day of hospitalization.

^ω^Anterior open bite.

^γ^Blood coagulation disorder with indication of suprapatellar amputation of lower limb.
