# Supplementary material for: Impact of achondroplasia on Latin American patients: a systematic review and meta-analysis of observational studies
Source: Orphanet J Rare Dis. 2022 Jan 4;17:4. doi: 10.1186/s13023-021-02142-3 (PMC8728937; doi:10.1186/s13023-021-02142-3)
Supplement: Supplementary file 4 — Additional file 4. Risk of bias for cross-sectional studies. [file 13023_2021_2142_MOESM4_ESM.docx]

**Supplementary Table 4.** Risk of bias for cross-sectional studies.

| Author, year | Were the aims of the study clear? | Was the study design appropriate for the stated aims? | Was the sample size justified? | Was the population clearly defined? | Was the sample frame taken from an appropriate population? | Was the selection process likely to select subjects that were representative of the population? | Were measures undertaken to address non-responders? | Were the risk factor and outcomes measured appropriate to the stated aims? | Were the risk and outcomes measured correctly using instruments that had been validated previously? | Is it clear what was used to determined statistical significance? | Were the methods (including statistical methods) sufficiently described to enable them to be repeated? | Were the demographic data adequately described? |
| --- | --- | --- | --- | --- | --- | --- | --- | --- | --- | --- | --- | --- |
| Arita et al., 2012^£^ [40] | Definitely yes | Definitely yes | Definitely no | Definitely yes | Definitely yes | Definitely yes | Unclear | Definitely yes | Definitely yes | Partially yes | Definitely no | Definitely yes |
| Barbosa-Buck et al., 2012^£^ [6] | Definitely yes | Definitely yes | Definitely yes | Definitely yes | Definitely yes | Definitely yes | Unclear | Definitely yes | Definitely yes | Definitely yes | Definitely yes | Definitely yes |
| Ceroni et al., 2018 [5] | Definitely yes | Definitely yes | Definitely no | Definitely yes | Definitely yes | Definitely yes | Unclear | Definitely yes | Unclear | Definitely no | Definitely no | Definitely no |
| Cervan et al., 2008^£^ [24] | Definitely yes | Definitely yes | Definitely no | Definitely yes | Definitely yes | Definitely yes | Unclear | Definitely yes | Definitely yes | Definitely yes | Definitely yes | Definitely yes |
| Escobar, 2014 [37] | Definitely yes | Definitely yes | Definitely no | Definitely yes | Definitely yes | Definitely yes | Unclear | Partially yes | Partially yes | Definitely no | Definitely no | Partially yes |
| Fano et al., 2000 [56] | Definitely yes | Definitely yes | Definitely yes | Definitely yes | Definitely yes | Definitely yes | Unclear | Definitely yes | Definitely yes | Definitely no | Definitely no | Definitely yes |
| Gomez et al., 2017 [58] | Definitely yes | Definitely yes | Definitely no | Definitely yes | Unclear | Unclear | Unclear | Unclear | Unclear | Definitely no | Definitely no | Definitely no |
| Junior et al., 2014 [29] | Definitely yes | Definitely yes | Definitely no | Definitely yes | Unclear | Unclear | Unclear | Unclear | Unclear | Unclear | Unclear | Definitely no |
| Lima, 2019 [38] | Definitely yes | Definitely yes | Definitely no | Definitely yes | Unclear | Unclear | Unclear | Definitely no | Definitely no | Definitely no | Definitely no | Definitely no |
| Medeiros et al., 2017; Medeiros et al., 2019 [76,77] | Definitely yes | Definitely yes | Definitely no | Definitely yes | Unclear | Definitely yes | Unclear | Definitely yes | Definitely yes | Definitely no | Definitely no | Definitely yes |
| Petitto & Baumotte, 2014 [67] | Definitely yes | Definitely yes | Definitely no | Definitely yes | Definitely yes | Unclear | Unclear | Definitely yes | Definitely yes | Definitely no | Definitely no | Definitely yes |
| Rocha & Wagner, 2018 [70] | Definitely yes | Definitely yes | Definitely no | Definitely yes | Unclear | Unclear | Unclear | Definitely yes | Definitely yes | Definitely no | Definitely no | Definitely no |
| Rodriguez-Gomez et al., 2015 [71] | Definitely yes | Definitely yes | Definitely yes | Definitely yes | Definitely yes | Definitely yes | Definitely yes | Definitely yes | Definitely yes | Definitely no | Definitely no | Partially yes |
| Sanchez et al., 1991 [73] | Definitely yes | Definitely yes | Definitely yes | Definitely yes | Definitely yes | Definitely yes | Unclear | Definitely no | Unclear | Partially yes | Definitely no | Definitely no |

Definitely yes = low risk of bias; partially yes = probably low risk of bias; partially no = probably high risk of bias; definitely no = high risk of bias; unclear = not enought information for a judgment.

^£^Comparative cross-sectional study.
