# Supplementary material for: Impact of achondroplasia on Latin American patients: a systematic review and meta-analysis of observational studies
Source: Orphanet J Rare Dis. 2022 Jan 4;17:4. doi: 10.1186/s13023-021-02142-3 (PMC8728937; doi:10.1186/s13023-021-02142-3)
Supplement: Supplementary file 5 — Additional file 5. Risk of bias for case series studies. [file 13023_2021_2142_MOESM5_ESM.docx]

**Supplementary Table 5.** Risk of bias for case series studies.

| Author, year | Were patient’s demographic characteristics clearly described? | Was the patient’s history clearly described and presented as a timeline? | Was the clinical condition of the patient clearly described? | Were diagnostic tests or assessment methods and the results clearly described? | Was the intervention(s) or treatment procedure(s) clearly described? | Was the post-intervention clinical condition clearly described? | Were adverse events identified and described? | Does the case report provide takeaway lessons? |
| --- | --- | --- | --- | --- | --- | --- | --- | --- |
| Alves, 2018 [34] | Partially yes | Definitely no | Partially yes | Definitely yes | Definitely yes | Definitely yes | Definitely yes | Definitely yes |
| Cervantes, 2006 [35] | Partially yes | Definitely no | Partially yes | Definitely yes | Definitely yes | Partially yes | Unclear | Definitely yes |
| Dantas et al & Medeiros, 2016 [26] | Unclear | Unclear | Unclear | Unclear | Unclear | Unclear | Unclear | Unclear |
| Tello et al., 1999 [74] | Partially yes | Partially yes | Definitely yes | Partially yes | Partially yes | Definitely no | Partially yes | Partially yes |

Definitely yes = low risk of bias; partially yes = probably low risk of bias; partially no = probably high risk of bias; definitely no = high risk of bias; unclear = not enought information for a judgment.
